# Direct Cardiac Actions of Sodium Glucose Cotransporter 2 Inhibitors Target Pathogenic Mechanisms Underlying Heart Failure in Diabetic Patients

**DOI:** 10.3389/fphys.2018.01575

**Published:** 2018-11-21

**Authors:** Laween Uthman, Antonius Baartscheer, Cees A. Schumacher, Jan W. T. Fiolet, Marius C. Kuschma, Markus W. Hollmann, Ruben Coronel, Nina C. Weber, Coert J. Zuurbier

**Affiliations:** ^1^Laboratory of Experimental Intensive Care and Anesthesiology, Amsterdam UMC, University of Amsterdam, Meibergdreef, Amsterdam, Netherlands; ^2^Clinical and Experimental Cardiology, Amsterdam UMC, University of Amsterdam, Meibergdreef, Amsterdam, Netherlands; ^3^IHU Liryc, Electrophysiology and Heart Modeling Institute, Fondation Bordeaux Université, Bordeaux, France

**Keywords:** SGLT2 inhibitors, cardiomyocyte, endothelial cell, smooth muscle cell, cardiac fibroblast, isolated heart, 2 type diabetes, heart failure

## Abstract

Sodium glucose cotransporter 2 inhibitors (SGLT2i) are the first antidiabetic compounds that effectively reduce heart failure hospitalization and cardiovascular death in type 2 diabetics. Being explicitly designed to inhibit SGLT2 in the kidney, SGLT2i have lately been investigated for their off-target cardiac actions. Here, we review the direct effects of SGLT2i Empagliflozin (Empa), Dapagliflozin (Dapa), and Canagliflozin (Cana) on various cardiac cell types and cardiac function, and how these may contribute to the cardiovascular benefits observed in large clinical trials. SGLT2i impaired the Na^+^/H^+^ exchanger 1 (NHE-1), reduced cytosolic [Ca^2+^] and [Na^+^] and increased mitochondrial [Ca^2+^] in healthy cardiomyocytes. Empa, one of the best studied SGLT2i, maintained cell viability and ATP content following hypoxia/reoxygenation in cardiomyocytes and endothelial cells. SGLT2i recovered vasoreactivity of hyperglycemic and TNF-α-stimulated aortic rings and of hyperglycemic endothelial cells. Anti-inflammatory actions of Cana in IL-1β-treated HUVEC and of Dapa in LPS-treated cardiofibroblast were mediated by AMPK activation. In isolated mouse hearts, Empa and Cana, but not Dapa, induced vasodilation. In ischemia-reperfusion studies of the isolated heart, Empa delayed contracture development during ischemia and increased mitochondrial respiration post-ischemia. Direct cardiac effects of SGLT2i target well-known drivers of diabetes and heart failure (elevated cardiac cytosolic [Ca^2+^] and [Na^+^], activated NHE-1, elevated inflammation, impaired vasorelaxation, and reduced AMPK activity). These cardiac effects may contribute to the large beneficial clinical effects of these antidiabetic drugs.

## SGLT2 Inhibitors in Diabetes and Heart Failure: Exploring Direct Cardiac Effects

Sodium glucose cotransporter 2 (SGLT2) inhibitors (SGLT2i) are kidney-targeted anti-diabetic agents that have exhibited marked reductions in cardiovascular events and mortality of type 2 diabetes (T2D) patients. In two large clinical trials, Empa, (Zinman et al., 2015) and Cana, (Neal et al., 2017) reduced the risk of heart failure related hospitalization by 35 and 33%, respectively. These trials demonstrate that SGLT2i may serve as an effective treatment strategy against heart failure in a T2D setting. SGLT2i were designed to inhibit the kidney-specific SGLT2 and to induce glycosuria. SGLT2 is part of a family of proteins facilitating glucose translocation in a variety of tissues. SGLT2 is mainly expressed in the kidney, with some expression in the pancreatic alpha cells. In the kidney, SGLT2 is located in the first part of the proximal tubule, where it enables ∼90% of glucose reabsorption from the urine; the other 10% reabsorption is through SGLT1, which is located in the distal part of the proximal tubule. SGLT2i use is associated with ∼50 g/day of glucose loss, which corresponds with ∼200 kcal/day (Ferrannini et al., 2015). Indeed, administration of a SGLT2i on top of standard glucose lowering therapy (metformin, insulin and sulfonylureas) modestly reduced (∼0.4%) glycated hemoglobin plasma levels and was associated with small decreases in body weight, plasma insulin and blood pressure (Zinman et al., 2015 Neal et al., 2017). However, it is unlikely that the small changes in these parameters can explain the large beneficial actions of SGLT2i, and thus preclinical studies were sought to explore possible underlying mechanisms.

*In vivo* preclinical studies have shown decreased ROS levels, inflammatory cytokines and vascular dysfunction after SGLT2i administration in animals with diabetes (Oelze et al., 2014; Andreadou et al., 2017; Tanajak et al., 2018). Moreover, preserved cardiac function has been observed with administration of SGLT2i Empa in a non-diabetic model of chronic heart failure (Byrne et al., 2017). In microvascular coronary endothelial cells of mice with STZ-induced diabetes, Empa administration resulted in activation of AMPK, improved mitochondrial function through inhibition of Drp1-mediated fission, restoration of vascular barrier function, eNOS phosphorylation, adhesion molecules expression and reduced mitochondrial ROS levels (Zhou et al., 2018). However, the *in vivo* animal studies cannot discern whether the cardiovascular beneficial effects of Empa and other SGLT2i are due to kidney-related systemic alterations or due to direct cardiovascular effects, or both. To examine whether SGLT2i operate directly on cardiac specific pathophysiological mechanisms without interference of other mediating factors, including plasma circulating glucose and insulin, isolated cardiac cell and organ studies are required. In this review we summarize the results of these studies and discuss how these direct cardiac effects of the inhibitors may contribute to the effects seen on heart failure. We restrict ourselves to cardiac cellular studies using clinically relevant SGLT2i concentrations (≤10 μM).

## Pathogenesis Linking Diabetes and Heart Failure

T2D is associated with a two to five fold higher risk of developing heart failure (Kannel et al., 1974; Cavender et al., 2015). Vice versa, chronic heart failure patients have an increased risk of developing T2D and metabolic abnormalities, including increased insulin resistance (Riehle and Abel, 2016). Thus, the presence of one disease impacts the development or progression of the other. Patients with heart failure and T2D have a worse prognosis than those with either one of the diseases (Cubbon et al., 2013).

**FIGURE 1:**
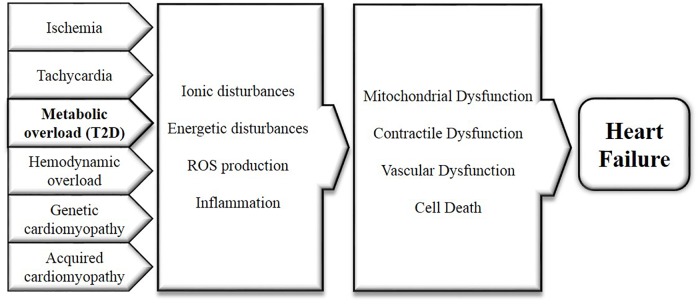
Mechanisms underlying and linking diabetes and heart failure, caused by cardiac metabolic overload and all other diseases with elevated risk for developing heart failure. These mechanisms include the disturbances of cellular ion levels, energetic disturbances, excessive ROS production and inflammation. Consistent dysregulation of these factors will sequentially instigate contractile, endothelial and mitochondrial dysfunction as well as cell death, as observed in the pathogenesis of heart failure.

Heart failure is a syndrome characterized by fatigue, dyspnea and the inability to exercise. It often develops after MI, valvular disease, chronic tachycardia, hypertension, diabetic cardiomyopathy, or in the setting of various genetic or acquired cardiomyopathies. The initial remodeling process associated with heart failure primarily includes left ventricular hypertrophy, fibrosis and diastolic dysfunction. Although the exact causes of T2D are still debated, hyperinsulinemia, insulin resistance, hyperglycemia and dyslipidemia are considered early contributors to the development of T2D (Jia et al., 2018). In T2D patients, insulin resistance and increased salt sensitivity lead to hypertension by increased sodium retention, activation of sympathetic nervous system and atherosclerosis (Feldstein, 2002; Lastra et al., 2010). Atherosclerosis in turn may cause MI and heart failure. Furthermore, the T2D heart is considered to be in a state of metabolic overload, with elevated glycogen and lipid stores within the heart (Varma et al., 2018). T2D is only one of several diseases that can develop into heart failure (see above). Early on in the development of heart failure, there are several key mechanisms that instigate functional and structural cardiac impairments, which are shared among heart failure and all these diseases, including T2D (Bugger and Abel, 2014; Packer, 2017). Therefore, targeting these early overlapping mechanisms is a promising strategy to treat heart failure. Several of these mechanisms with potential relevance to direct myocardial effects of SGLT2i are summarized in Figure 1.

First, elevated cytosolic [Na^+^] and [Ca^2+^] ([Na^+^]_c_ and [Ca^2+^]_c_) are observed in cardiomyocytes from failing and T2D hearts (Despa et al., 2002; Lambert et al., 2015; Despa, 2018). Elevations in [Na^+^]_c_ and [Ca^2+^]_c_ are coupled to reduced mitochondrial [Ca^2+^] ([Ca^2+^]_m_) in cardiomyocytes, which will decrease the energetic and redox function of mitochondria (Murphy and Eisner, 2009; Bay et al., 2013). Elevated [Ca^2+^]_c_ is perceived in hyperglycemia- and LPS-induced endothelial dysfunction (Wang et al., 2008; Cui et al., 2013), atrial fibrillation (Neef et al., 2010) and in myocytes during IR injury (Garcia-Dorado et al., 2012). Increases in [Na^+^]_c_ and [Ca^2+^]_c_ are caused by perturbed ion fluxes due to altered activity of ion channels and/or transporters, including the sodium-calcium exchanger (NCX), the sodium-hydrogen exchanger 1 (NHE-1), the ryanodine receptor regulating SR-calcium release and the sodium-potassium ATPase (Na^+^/K^+^ ATPase), and targeting these ion regulating transporters has been proposed to improve cardiovascular function (Baartscheer et al., 2003a; Despa et al., 2012; Karmazyn, 2013; Luo et al., 2013; Sasahara et al., 2013).

Second, the development of an energy crisis, best reflected by decreases in cardiac PCr/ATP, is present in both heart failure and diabetic cardiomyopathy. As a consequence, the cellular energy sensor AMPK is altered. AMPK activity is increased in failing myocytes (Tian et al., 2001), whereas in T2D divergent AMPK alterations have been found (Varma et al., 2018). AMPK acts as an energy sensor, and activates catabolic actions such as increasing glucose uptake and glycolysis, and impairing anabolic reactions (Beauloye et al., 2011). Targeting the AMPK pathway by increasing its activity have been shown to combat cardiac hypertrophy and heart failure (Gélinas et al., 2018). Besides, AMPK activity is relevant in non-metabolic cellular processes, including the regulation of vascular tone and the suppression of inflammation (Beauloye et al., 2011; He et al., 2015; Cordero et al., 2018). Metformin, the first-line drug in the treatment of T2D for the last two decades, is known to increase AMPK (Foretz et al., 2014), an effect that can also be observed in the human non-diabetic heart tissue (El Messaoudi et al., 2015).

Third, early increased oxidative stress figures prominently in both heart failure (Dey et al., 2018) and T2D pathogenesis (Shah and Brownlee, 2017). The highly reactive ROS can oxidize many proteins in its vicinity, with consequently altered function of that protein. ROS is also an upstream driver of endothelial dysfunction associated with heart failure, by the increase of nitric oxide synthase (NOS) uncoupling, switching the production of NO into that of peroxynitrite (Paulus and Tschöpe, 2013; Joshi et al., 2014; Münzel et al., 2015). Reducing ROS therefore will normalize the function of many proteins of cardiac cells and may so undermine the development of heart failure.

Fourth a chronic low-grade state of inflammation is present in both heart failure and diabetes. Cellular ion dysregulation, AMPK inactivity and ROS production are relevant factors in the development of inflammation (Frati et al., 2017). Other mechanisms that underlie the progression of inflammation in diabetes may include increased *O*-GlcNAcylation, formation of AGEs, activation of the renin-angiotensin-aldosterone system, damage-associated molecular patterns (DAMPs) and the production of cytokines and adipokines (Frati et al., 2017). Inflammation may lead to fibrosis, cell death (pyroptosis) and cardiac remodeling, and its suppression may therefore be a relevant mechanism in the prevention and treatment of diabetes-associated heart failure.

These four mechanisms are not separate entities but are all intrinsically interrelated, and together may induce contractile, vascular and mitochondrial dysfunction, and cell death in the heart, which may evolve into left ventricular concentric or eccentric hypertrophy and heart failure (Boudina et al., 2007; Van Heerebeek et al., 2008; Miki et al., 2013; Sena et al., 2013; Cassidy et al., 2015; Munasinghe et al., 2016). In the subsequent paragraphs, SGLT2i effects in the major cardiac cell types will be discussed in relation to these four early signatures (ion homeostasis, energy balance, oxidative stress and inflammation) of both heart failure development and diabetic cardiomyopathy.

## SGLT2 Inhibitors in Cardiomyocytes

### Intracellular Ion Regulation

Several reports have documented that SGLT2i modify ionic homeostasis in cardiomyocytes. Hamouda et al. (2014) showed that 1–10 μM Dapa reduced the amplitudes of cell shortening and L-type Ca^2+^ current in ventricular cardiomyocytes from STZ-treated and control rats. Dapa only lowered systolic [Ca^2+^]_c_, but not diastolic [Ca^2+^]_c_, in STZ-treated rat cardiomyocytes, however, this effect was absent in control rat cardiomyocytes. These changes were observed at *t* = 5 min and were absent after 1–3 h of incubation with 1 μM Dapa (Hamouda et al., 2014). These data suggest that Dapa may confer acute negative inotropic effects in the diabetic cardiomyocyte. In support of this, Empa (0.25–1 μM) reduced [Na^+^]_c_, diastolic and systolic [Ca^2+^]_c_, and impaired the NHE-1 activity in healthy rabbit cardiomyocytes (Baartscheer et al., 2017). In that study, Empa also increased [Ca^2+^]_m_ in rat cardiomyocytes. This finding may reflect improved mitochondrial capacity to synthesize ATP and target oxidants, which would be beneficial to restore the energetic state of myocytes that is known to be decreased in heart failure. The lowering of [Na^+^]_c_ by Empa was observed at 5 mM and 11 mM glucose incubations and inhibition of NHE-1 occurred in the presence and absence of extracellular glucose, supporting the notion that glucose and SGLT’s were not involved in these direct Empa effects. In addition, we recently showed that Dapa (1 μM) and Cana (3 μM) also reduced [Na^+^]_c_ and impaired NHE-1 activity in mouse cardiomyocytes (Uthman et al., 2018a). Further prove for SGLT2i interaction with NHE-1 was obtained by molecular binding studies, showing SGLT2i to exhibit high binding affinities with the extracellular Na^+^-binding site of the NHE (Uthman et al., 2018a). These data indicate that the SGLT2i exert an off-target effect on the NHE-1.

### Mitochondrial Function

Empa increased cell viability and preserved ATP levels following hypoxia/reoxygenation in cultured H9C2 embryonic heart-derived cells (Andreadou et al., 2017). These effects were equally present in myocytes stimulated by AGE, and Empa did not change the expression level of RAGE (receptor for AGE), suggesting that these pro-survival mechanisms of Empa were not mediated through AGE/RAGE signaling. In mitochondria derived from cardiac muscle fibers obtained from isolated IR rat hearts, Empa increased complex II respiration, and permeabilized and uncoupled the inner mitochondrial membrane (Jespersen et al., 2017). The authors discuss that these mitochondrial changes may improve remodeling following IR conditions.

### Expression of SGLT’s

Although the SGLT2 has not been detected in cardiomyocytes and the heart, SGLT1 appears to be highly expressed in human, rat and mouse hearts and cardiomyocytes, and may even be upregulated in ischemic, hypertrophic, failing and diabetic hearts (Banerjee et al., 2009; Kashiwagi et al., 2015; Lambert et al., 2015; Vrhovac et al., 2015; Di Franco et al., 2017; Van Steenbergen et al., 2017). Currently, the relationship between SGLT2i and SGLT1 in failing and diabetic hearts has not been investigated.

**FIGURE 2:**
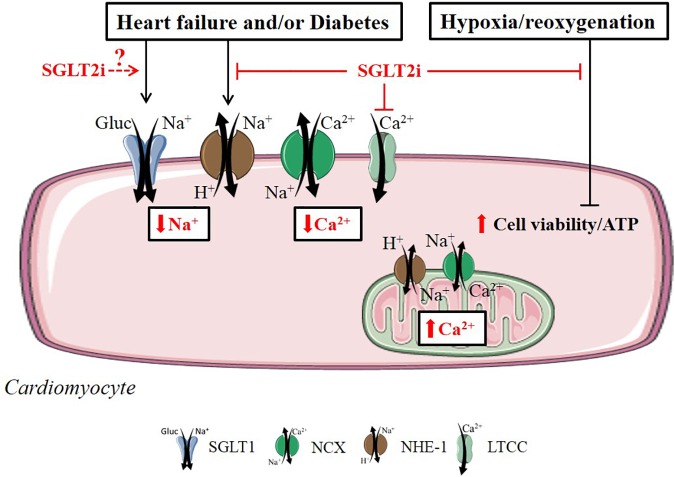
Direct SGLT2i effects related to diabetes, heart failure and hypoxia/reoxygenation in the cardiomyocyte. SGLT2i exert beneficial cardiac cell effects in by directly regulating ionic homeostasis, mitochondrial respiration and cell viability in the cardiomyocyte. Reported intracellular SGLT2i effects are indicated in red arrow symbols. The relationship between SGLT2i and cardiac SGLT1 in the diabetic or failing cardiomyocyte is unknown (illustrated by dashed arrow line in the figure).

Overall, SGLT2i directly affect cardiomyocytes by reducing [Na^+^]_c_ and [Ca^2+^]_c_, by inhibition of NHE-1, and by increasing mitochondrial function and cell viability (Figure 2). The exact membrane receptors/transporters mediating these effects in cardiomyocytes have not yet been identified, with the exemption of NHE-1 and LTCC.

## SGLT2 Inhibitors in Vascular Cells

**FIGURE 3:**
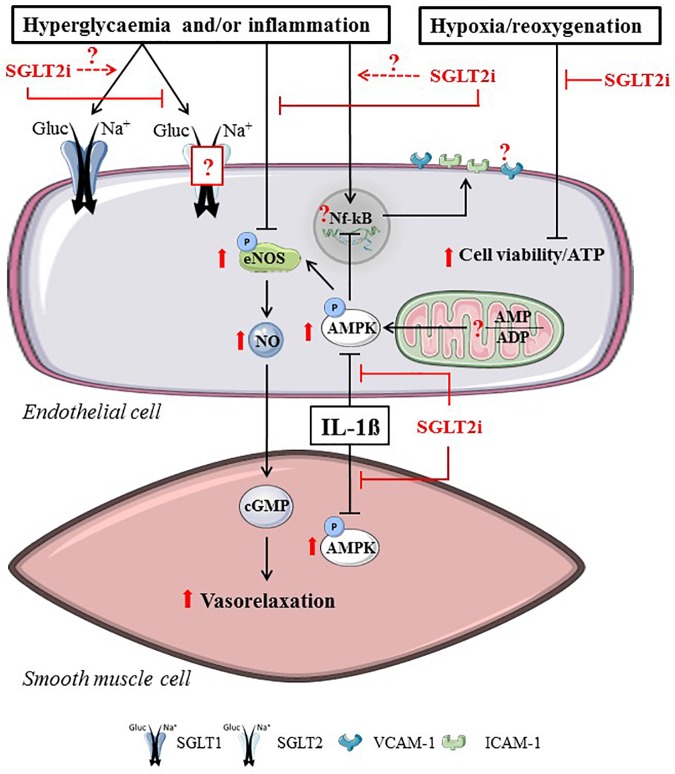
Direct SGLT2i effects related to hyperglycemia, inflammation or hypoxia/reoxygenation in vascular cells. SGLT2i directly alter endothelial cells and smooth muscle cells by reducing SGLT2-mediated glucose uptake, ameliorating vasorelaxation, increasing AMPK activity and maintaining cell viability in vascular cells. Reported intracellular SGLT2i effects are indicated in red arrow symbols. The presence of SGLT2 in endothelial cells, the interaction between SGLT1 and SGLT2i, the effects of SGLT2i on AMP/ADP and the attenuation of NF-kB-mediated adhesion molecule expression remains uncertain (illustrated by dashed arrow lines and question marks in the figure).

Vascular function may be directly affected by SGLT2i through the changing of endothelial or vascular smooth muscle cell (SMC) function. Various reports have investigated SGLT2i effects on AMPK activity, mitochondrial function, vasodilation and on the expression of adhesion molecules in hyperglycemic and inflamed vascular cells (Figure 3).

### AMPK Activity

Cell studies with SGLT2i in various disease models have generally shown that SGLT2i improve vascular function and/or reduce markers of atherogenesis, by directly acting on vascular cells. Increased AMPK activity was observed in intact HUVEC and HAEC, after 15–30 min treatment with Cana (≥10 μM) but not with Empa or Dapa (Mancini et al., 2018). Similarly, phosphorylation of ACC as an indicator of AMPK activation was increased acutely with 10 μM Cana in cultured human aortic vascular SMC (Mancini et al., 2018).

### Cell Viability and Mitochondrial Function

In HUVEC subjected to hypoxia/reoxygenation, Empa increased cell viability and preserved ATP levels in the presence and absence of AGE (Andreadou et al., 2017). In healthy cells, viability remained unaltered with 24 h Empa or Dapa treatment, but was modestly reduced with Cana treatment, which is suggested to be caused by reduced glucose uptake causing compromised ATP synthesis (Mancini et al., 2018). Empa (1 μM) did prevent mitochondrial dysfunction in hyperglycemic mouse aortic rings, i.e., increased oxygen consumption rates and reduced proton leakage (El-daly et al., 2018). It is uncertain, however, whether Empa would affect the vasculature at physiologically more relevant hyperglycemic glucose concentrations (<20 mM). Together, these data suggest that SGLT2i directly improve cell viability under conditions of high glucose, and that this may be due to mitochondrial alterations.

### Vasodilation

The effects of Dapa on endothelial cell- and SMC- dependent vascular tone in various models have been studied. Acute Dapa administration (1 nM–100 μM) increased vasorelaxation in an endothelial cell-independent manner in healthy mouse aortic rings, while in a hyperglycemic setting (44 mM glucose) 1 h pre-incubation with 1 μM Dapa restored relaxation without influencing SMC vasoreactivity (Gaspari et al., 2017). Acute treatment with high dose Cana (10 μM) caused vascular relaxation in pulmonary, but not coronary artery rings from single STZ-injected mice (Han et al., 2015), indicating vascular bed specific effects of SGLT2i. Pre-treatment with 0.5–1 μM Empa, 50 nM Dapa or 50 nM Cana for 24 h resulted in a stronger vasorelaxation response in hyperglycemic (25 mM glucose) mice aortic rings following application of acetylcholine or a proteinase-activated receptors 2 agonist (El-daly et al., 2018). Likewise, exposure of 1 μM Empa for 3 days promoted eNOS activity (nitrite formation) in hyperglycemic HUVEC, yet cell density was not restored at this physiologically relevant Empa dosage (Steven et al., 2017), suggesting that the functional improvement by Empa did not directly correlate to increased cell viability.

### Cell Adhesion

In IL-1β-stimulated HUVEC and HAEC, 10 μM Cana attenuated secretion of MCP-1 and IL-6, which was at least partly associated with increased AMPK activity (Mancini et al., 2018). Dapa treatment (1–1000 nM) resulted in lower ICAM-1 and VCAM-1 protein levels and NFκB mRNA expression in HUVEC subjected to TNF-α or to a hyperglycemic insult. However, low dose Dapa (≤10 nM), but not 100 nM and 1 μM Dapa, seemed to reduce the inflammatory responses from hyperglycemia or TNF-α (Gaspari et al., 2017). Knowing that the maximal plasma concentrations of Dapa healthy volunteers taking 10 mg Dapa fluctuates between ∼6–300 nM (Kasichayanula et al., 2011), it raises the possibility of Dapa having anti-inflammatory actions in the T2D and/or heart failure patient. Contrary to Dapa application, Mancini et al. (2018) reported that adhesion molecule expression, JNK and NFκB signaling of LPS-stimulated HAEC were not altered after Cana administration (Mancini et al., 2018), although adhesion to pro-monocytic cells was reduced. Our own results indicated that SGLT2i pre-incubation does not prevent ICAM and VCAM expression and the increase in permeability in TNF-α stimulated HUVEC and human coronary artery endothelial cells [HCAEC, (Uthman et al., 2018b)].

### Expression of SGLT’s

As several studies reported absence of SGLT2 expression in the heart (Han et al., 2015; Vrhovac et al., 2015; Di Franco et al., 2017; Van Steenbergen et al., 2017), SGLT2 may be excluded as a possible explanation for the direct vascular effects of SGLT2i. However, others have proposed that the SGLT2 mRNA is ubiquitously expressed in most human tissue (Zhou et al., 2003) and that the SGLT2 protein is also present in endothelial cells (El-daly et al., 2018; Li et al., 2018). In HUVEC, both SGLT1 and SGLT2 expressions were demonstrated at the protein and mRNA level and their quantities were elevated in the presence of PA, which also resulted in reduced Akt, IRS-1 and eNOS phosphorylation and nitric oxide concentration (Li et al., 2018). PA-induced endothelial dysfunction was partially restored with administration of the classic SGLT inhibitor phlorizin, and knockdown of SGLT1 or SGLT2. Additionally, Empa reduced glucose uptake in cultured endothelial cells similar to SGLT1/2 inhibitor sotagliflozin, measured by reduced glucose analog 2-NBDG uptake (El-daly et al., 2018). So far, no direct target of SGLT2i on endothelial cells has been identified, although the presence of SGLT1 and SGLT2 in endothelial cells might be connected to the direct vascular effects summarized above.

## SGLT2 Inhibitors in Cardiac Fibroblasts

**FIGURE 4:**
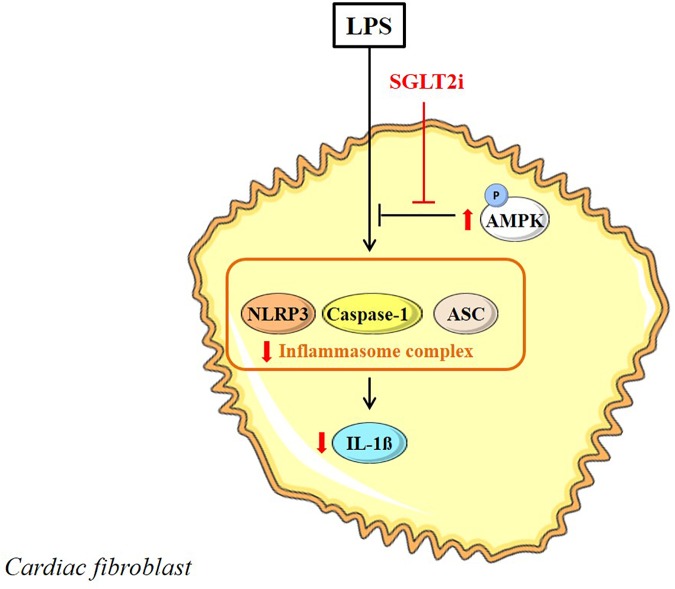
Direct SGLT2i in the LPS-stimulated myofibroblast. SGLT2i increase AMPK activity and inhibit inflammasome activation in the myofibroblast. Reported intracellular SGLT2i effects are indicated in red arrow symbols.

Cardiac fibroblasts are valuable targets for therapeutic applications due to their role in cardiac remodeling after MI. Ye et al. (2017) studied SGLT2i effects in mouse cardiac fibroblasts stimulated with LPS. Pre-incubation with Dapa (0.3–0.5 μM) showed attenuation of LPS-induced upregulation of NLRP3, ASC, IL-1β and caspase-1 mRNA levels (Ye et al., 2017). Administration of phlorizin (100 μM) did not exhibit similar reponses as Dapa and this indicates that SGLT-independent mechanisms were affected by Dapa. Effects of Dapa on NLRP3, TNF-α and caspase-1 were similar to A769662, an AMPK activator, while co-administration of Dapa with Compound C, an AMPK inhibitor, abrograted the anti-inflammatory effects, including phosphorylation of AMPKα at Thr172. Thus, Dapa induces anti-inflammatory responses in myofibroblasts, mediated through increased AMPK activation without the involvement of SGLT (Figure 4).

## SGLT2 Inhibitors in Isolated Hearts

Data retrieved from isolated heart studies indicate that SGLT2i have direct cardiac effects at organ level, i.e., acute vasodilation, delayed ischemic contracture onset and increased post-ischemic P-STAT3 expression. The degree of IR injury and vasodilation, however, relies upon the concentration administered as well as the type of SGLT2i.

### Vasodilation

Several studies have addressed the effects of SGLT2i in the isolated perfused heart. Acute coronary vasodilation occurs in intact mouse hearts following Empa (1 μM) or Cana (3 μM), but not after Dapa (1 μM) (Uthman et al., 2018a). These findings may reflect the differences in drug efficacy since Dapa did show a trend toward vasodilation, which may suggest that a higher concentration of Dapa could induce vasodilation in the healthy heart.

### Ischemia-Reperfusion

Empa (4.75 μM) administration prior to ischemia did not change infarct size after 40 min ischemia and 30 min reperfusion in the isolated rat heart (Jespersen et al., 2017). However, during a 25 min ischemia and 2 h reperfusion protocol in isolated mouse hearts, Empa (1–10 μM) delayed ischemic contracture development similar to the effects of a classic NHE-1 inhibitor Cariporide. Reperfusion injury remained unaltered or even worsened for the Empa-treated hearts under these conditions (Uthman et al., 2018c). These effects may indicate an ATP-sparing effect of SGLT2i that is caused by inhibition of NHE-1 during ischemia, although NHE-1 inhibition during reperfusion may differ between Empa and Cariporide. Furthermore, the detrimental outcomes of high-dosage Empa (higher than the maximal concentration observed in clinical trials), may possibly refer to potential cell toxic effects of this drug used at a higher concentration during IR injury. Furthermore, Empa did not change mitochondrial calcium retention capacity in mitochondria isolated from mouse hearts (Andreadou et al., 2017). This suggests that Empa does not directly affect the mitochondrial permeability transition pore, which is in line with findings reflecting that Empa does not protect the isolated heart against IR injury (Jespersen et al., 2017; Uthman et al., 2018c).

### Fibrosis

Perfusion with a high dose of Dapa (10 μM) for 60 min increased STAT3 phosphorylation in the remote area of post-ischemic rat hearts (Lee et al., 2017). Increased p-STAT3 levels were associated with higher IL-10 levels in Dapa treated hearts, which is suggested to facilitate the conversion of macrophages from an inflammatory M1 to anti-inflammatory M2 phenotype and therefore inhibit myofibroblast differentiation and extracellular matrix formation.

## Discussion

This review provides an overview of the direct effects of SGLT2i in the heart and in the most prominent cell types present in the heart, including cardiomyocytes, endothelial cells, vascular SMC and cardiofibroblasts. Figures 2–4 graphically summarize the described effects of SGLT2i in these cardiac cell types. They show that SGLT2i positively affect the Na^+^ and Ca^2+^ homeostasis, inhibit NHE-1 activity, improve mitochondrial respiration, and increase post-hypoxic ATP levels and cell viability in cardiomyocytes. In vascular cells, SGLT2i can directly improve vasorelaxation, increase AMPK activity, eNOS phosphorylation and, although inconsistently, adhesion molecule expression in hyperglycemic and/or inflamed conditions. In LPS-treated cardiofibroblasts, SGLT2i exert anti-inflammatory actions similar to AMPK activation pathways. Finally, in isolated hearts SGLT2i improved vasodilation, delayed ischemic contracture and increased post-ischemic STAT3 phosphorylation. These preclinical cell/organ studies clearly show that there are direct cardiac effects of SGLT2i, offering several off-target, cardioprotective mechanisms that may contribute to the beneficial effects observed in the clinical trials.

### Ionic Homeostasis in Diabetes and Heart Failure

A growing number of pre-clinical studies have demonstrated beneficial cardiovascular effects of SGLT2i independent of their systemic glucose-lowering actions (Andreadou et al., 2017; Baartscheer et al., 2017; Byrne et al., 2017; Ye et al., 2017; Uthman et al., 2018a). In light of these results, the direct lowering of [Na^+^]_c_ by SGLT2i may constitute part of the beneficial effects of SGLT2i through attenuation of inflammation, endothelial dysfunction, oxidative stress and cardiac remodeling.

To reduce the global cardiovascular risk, the World Health Organization advises to lower daily salt intake within the population to <5 g (WHO, 2012). High salt (i.e., Na^+^) intake evidently predicts the development of heart failure and diabetes (He and MacGregor, 2018) as well as an increased incidence of T2D in individuals with high caloric intake (Lanaspa et al., 2018). Na^+^ loading contributes to elevated oxidative stress and activation of the NLRP3 inflammasome (inflammation), consequently leading to the development of diabetes in humans (Wan et al., 2018). In endothelial cells, a mild increase of extracellular Na^+^ led to elevated levels of adhesion molecules and the transmigration of peripheral blood mononuclear cells (Dmitrieva and Burg, 2015). An exact molecular mechanism of Na^+^ loading that causes inflammation has not been experimentally determined, although it has been reported that the efflux of K^+^ via the Na^+^/K^+^-ATPase is a necessary step that triggers NLRP3 activation (Muñoz-Planillo et al., 2013). Increased Na^+^/K^+^-ATPase through elevated extracellular Na^+^ may therefore cause K^+^ efflux and activate NLRP3. Notably, increased extracellular Na^+^ also led to activation of NLRP3 independent of K^+^ efflux (Muñoz-Planillo et al., 2013). Taken together, extracellular Na^+^ is an important determinant of inflammation and may therefore be relevant in targeting cardiovascular disorders.

A modest increase in extracellular glucose (from 5.5 to 11 mM) acutely elevated [Na^+^]_c_ in isolated cardiomyocytes (Baartscheer et al., 2017). Chronic hyperglycemia, as occurring in T2D and heart failure, may thus at least partly be accountable for the increase in cardiac [Na^+^]_c_. Increased [Na^+^]_c_ at a steady extracellular glucose concentration was recently observed in T2D rat cardiomyocytes, which could be ascribed to elevated Na^+^-glucose cotransport through increased expression of SGLT1 (Lambert et al., 2015). Cardiac SGLT1 expression is increased in conditions of T2D and heart failure, in both animal and man (Banerjee et al., 2009; Lambert et al., 2015; Di Franco et al., 2017). Thus, [Na^+^]_c_ in cardiomyocytes can be increased due to increased extracellular glucose levels as well as increased SGLT1 expression.

Elevation of [Na^+^]_c_ results in a secondary rise of [Ca^2+^]_c_ via the Na^+^/Ca^2+^-exchanger (NCX) (Baartscheer et al., 2003a). Studies support the contention that increases in [Na^+^]_c_ and [Ca^2+^]_c_ induce signaling cascades that lead to dysregulation of mitochondrial homeostasis, including impaired energetics and elevated ROS production, and as such reduced cardiac hypertrophy and remodeling [reviewed in detail elsewhere: (Bay et al., 2013)].

The reduction in [Ca^2+^]_c_ by SGLT2i appears to be directly correlated to the lowering of [Na^+^]_c_. The [Na^+^]_c_ lowering effects of SGLT2i can be explained by inhibition of NHE-1. Cardiomyocytes express high amounts of NHE-1 (Yokoyama et al., 2000), which is also of paramount importance for pH regulation during pathological conditions, including diabetes, IR injury and heart failure (ten Hove et al., 2003; Packer, 2017). Preclinical studies have shown that pharmacological interventions targeting sarcolemmal sodium ion transporters proved effective in ameliorating heart failure (Kusumoto et al., 2001; Engelhardt, 2002; Baartscheer et al., 2003b, 2005, 2008; Baartscheer and Van Borren, 2008). However, these promising preclinical studies in the setting of HF on the use of NHE-1 inhibition never translated into clinical testing for HF, because of negative results obtained with the use of these inhibitors in the setting of acute cardiac ischemia. Clinical trials on the short-term use (<7 days) of NHE-1 inhibitors in the setting of acute MI in CABG and PTCA patients showed a reduction in the incident of non-fatal MI without an overall benefit on cardiovascular mortality. These clinical studies were halted due to the occurrence of an increased incidence in cerebrovascular events in these acute cardiovascular conditions following treatment with the NHE-1 inhibitors [Control vs. NHE inhibitor; EXPEDITION 3.0% vs. 5.2% (*p* < 0.001); GUARDIAN-trial 1.0% vs. 1.5%; ESCAMI-trial 0.2 vs. 2.2% (not significant)] (Théroux et al., 2000; Zeymer et al., 2001; Mentzer et al., 2008). In addition, the optimal dosage, timing and duration of the intervention and the patient populations that could most likely benefit from NHE-1 inhibitors were insufficiently investigated by phase 2 clinical trials, which may explain the divergence between the highly promising preclinical results of NHE-1 inhibitors and the ambiguity hereof in the known clinical trials. Furthermore, it is known that mild acidosis, but not severe acidosis, may be protective against ischemia in neuronal tissue (Vornov et al., 1996). Most importantly, to date no clinical trial has investigated the effects of using NHE-1 inhibitors as chronic therapy for the treatment of heart failure. Possibly, the risk of stroke may be less pronounced or even absent if NHE-1 inhibitors are used chronically, not immediately halted, used at milder dosages and in combination with platelet inhibitors. Therefore, the clinical efficacy of inhibiting NHE-1 and lowering [Na^+^]_c_ in different chronic cardiac disorders remains to be tested.

Previous experiments with Empa have shown that NHE-1 inhibition also occurred in glucose-free conditions, thus eliminating SGLT’s as mediators for the lowering of [Na^+^]_c_ (Baartscheer et al., 2017). Whether SGLT2i also inhibit NHE-1 activity in diabetic cardiomyocytes has not yet been investigated, although increased activity of NHE-1, accompanied by higher [Ca^2+^]_c_ and hypertrophy has been observed in the diabetic myocardium (Darmellah et al., 2007). Others have reported elevated NHE-1 expression in glucose-induced hypertrophy in isolated rat cardiomyocytes, which may be prevented by treatment with Cariporide (Chen et al., 2007).

In diabetic and failing hearts, the cellular energetics (PCr/ATP) are impaired (Beer et al., 2002; Levelt et al., 2016). ATP generation is directly linked to [Ca^2+^]_m_ as it determines the regulation of dehydrogenases, generation of reducing equivalents for oxidative phosphorylation, Δ*G*_ATP_ and the inner mitochondrial membrane potential (Glancy and Balaban, 2012). It has been demonstrated that [Ca^2+^]_m_ is reduced when [Na^+^]_c_ is elevated from 5 to 15 mM as the exchange of [Ca^2+^]_m_ and [Na^+^]_c_ through the mitochondrial NCX is raised (Liu and Rourke, 2008). Conversely, Empa led to elevated [Ca^2+^]_m_ in myocytes (Baartscheer et al., 2017), probably because of reduced exchange of [Na^+^]_c_ for [Ca^2+^]_m_ due to lower [Na^+^]_c_. The increased [Ca^2+^]_m_ is likely to increase cardiac energetics resulting in an increased PCr/ATP ratio (Bertero and Maack, 2018). Although in healthy hearts SGLT2i were without effect on PCr/ATP levels (Uthman et al., 2018a), it cannot be excluded that SGLT2i will increase PCr/ATP levels in diabetic and failing myocytes, especially since it is already observed that ATP levels are preserved in cultured cardiomyocytes subjected to hypoxia/reoxygenation (Andreadou et al., 2017).

### AMPK Activation to Improve Energetics and Prevent Inflammation

5′ AMPK is a regulator of cardiac energy metabolism. Given that myocardial energetic status is severely compromised in failing (Neubauer, 2007; Ingwall, 2009) and diabetic (Levelt et al., 2016) hearts, AMPK activation could enhance cardiac energy metabolism and hence restore myocardial energy levels (Beauloye et al., 2011). Furthermore, activation of AMPK reduced inflammation and oxidative stress levels in PA-stimulated endothelial cells (Li et al., 2015). Cana in endothelial cells and Dapa in myofibroblasts activated AMPK, resulting in reduced inflammatory responses in these cells (Ye et al., 2017; Mancini et al., 2018). A possible mechanism by which SGLT2i change AMPK activity is through complex I inhibition and increased AMP/ADP ratio, which was examined in mouse hepatocytes (Hawley et al., 2016). However, this effect was only observed with high doses of Cana, and not with Empa or Dapa. One may also postulate that the inhibition of glucose uptake by SGLT2 inhibition may activate AMPK as a compensatory feedback mechanism to restore cell metabolism. Limited data are available as of yet that explains how SGLT2i could activate AMPK. Further research with, e.g., knock-down models of SGLT2 could elaborate on the cellular mechanism of SGLT2i to directly activate AMPK.

Whether the SGLT2i convey through similar AMPK activation pathways in cardiac cells needs further investigation. Based on the existing research, lower dosages of Dapa (≤0.5 μM) seemed to be most effective for AMPK activation and the suppression of inflammation, while higher Cana concentrations activated AMPK and reduced inflammatory responses in AMPK-dependent and -independent manners. These results imply that the different SGLT2i activate AMPK at different concentrations.

### Inflammation and Oxidative Stress

Inflammation is considered an essential driving factor of cardiovascular disease in diabetes, whereas elevated levels of extracellular glucose alone is insufficient to induce a pro-atherogenic state (Sharma et al., 2018). Instead, the combination of glucotoxic and inflammatory stimuli are needed to establish an environment prone to developing atherosclerosis (Azcutia et al., 2010). Current understandings of the pathophysiology of heart failure, in particular with preserved ejection fraction (HFpEF) postulate that the presence of comorbidities cause microvascular inflammation that ultimately leads to the development of heart failure (Paulus and Tschöpe, 2013). Importantly, vascular inflammation can be induced by reduced AMPK activity (He et al., 2015) as well as high Na^+^ loading, the latter resulting in elevation of fasting blood glucose levels, oxidative stress and insulin resistance (Wan et al., 2018). Endothelial inflammation then leads to perturbed NO-cGMP-PKG signaling and increased leukocyte trafficking, which subsequently induces cardiomyocyte hypertrophy and myofibroblast differentiation, and as such cardiac remodeling. Since SGLT2i have demonstrated direct anti-inflammatory actions in vascular cells, targeting this new paradigm may potentially and at least partly explain the positive results observed in the clinical trials and may identify novel therapeutic implications for SGLT2i.

### A Window for SGLT2i as Anti-arrhythmic Therapy

In heart failure, cardiac arrhythmias primarily depend on triggered activity (Coronel et al., 2013). Elevated levels of diastolic [Ca^2+^]_c_ increase the open probability of the Ryanodine receptor resulting in spontaneous release of calcium from the SR. This in turn activates the NCX, leading to the transient inward current responsible for delayed after depolarizations (Baartscheer et al., 2003c). In addition, it has been shown that the number of after-transients measured in isolated myocytes is related to the occurrence of ventricular tachycardia *in vivo* (Janse et al., 2001). No data are available on the effects of SGLT2i on arrhythmogenesis. However, under pathologic conditions where myocardial [Na^+^]_c_ is increased, such as in heart failure, a reduction of [Na^+^]_c_ is associated with a reduction of [Ca^2+^]_c_, the number and amplitude of Ca^2+^ after-transients and the associated arrhythmias (Despa et al., 2002; Pogwizd et al., 2003; Bers, 2014). In the same vein of thought, we speculate that the inhibitory effect of SGLT2i on Na^+^ influx during heart failure is antiarrhythmic.

Erickson et al. (2013) have documented that Ca^2+^/calmodulin-dependent kinase II (CaMKII) is elevated in diabetes and that it is associated with increased Ca^2+^-release events from the SR. Thus, diabetes directly impacts arrhythmogenesis. Recent work by Mustroph et al. (2018) has demonstrated that Empa reduced CAMKll activity and CaMKII-dependent SR Ca^2+^ leak. Thus, Empa is likely to directly reduce Ca^2+^-release and arrhythmias. In addition, indirect effects of SGLT2i on arrhythmogenesis can be mediated through attenuation of the cardiac remodeling and hypertrophic phase known to occur in diabetes (Byrne et al., 2017).

### The Presence of SGLT2 in Endothelial Cells?

Given that several studies have reported that SGLT2i directly reduced glucose uptake in endothelial cells, it may be possible that this occurred as a result of SGLT2 inhibition. Conflicting results regarding the expression of SGLT2 in the coronary vasculature have been reported. While SGLT2 has not been detected at all in the heart (Van Steenbergen et al., 2017), increasing evidence demonstrate the existence of SGLT2 in non-cardiac endothelial cells (El-daly et al., 2018; Li et al., 2018). Existing data that suggested the absence of SGLT2 in the endothelium had only attempted to detect SGLT2 at mRNA levels. However, more recent studies have postulated the presence of SGLT2 in endothelial cells at protein level and that its expression levels are amendable by exposure to SGLT2-specific siRNA or PA (El-daly et al., 2018; Li et al., 2018), providing further support for a novel mechanism of SGLT2 and its inhibitors in vascular cells. A major limitation for the identification of SGLT2 at protein level is the lack of knowledge on the quality and specificity of the antibodies used. Therefore, further development of these techniques are highly warranted. Whether diabetic or failing conditions may evoke SGLT2 expression in cardiac endothelial cells has so far not been investigated.

### SMIT-1 as Potential Target for SGLT2i

Another possible target for SGLT2i is the sodium-myoinositol cotransporter 1 (SMIT-1), a member of the SGLT receptor family. Overexpression of SMIT-1 leads to activation of NOX2, ROS production and increased glucose sensitivity in cardiomyocytes, and its deletion triggered opposite effects (Van Steenbergen et al., 2017). The effects of increased SMIT-1 expression were not associated with increased glucose uptake with higher extracellular glucose concentration, and the authors suggest that SMIT-1 effects relate to extended glucose sensitization that could alter downward signaling events related to [Na^+^]_c_ and [Ca^2+^]_c_. Considering the [Na^+^]_c_ lowering effect of SGLT2i, SGLT2i targeting SMIT-1 during hyperglycemic condition to attenuate hyperglycemia-induced damage might be assumed. However, Baartscheer et al. (2017) showed Na^+^-lowering with Empa, even in the absence of glucose. Furthermore, the IC50 of Empa and Cana for SMIT-1 were estimated at 8.3 μM and 5.6 μM, respectively, which is far off the *C*_max_ for Empa (0.6 μM) (Suzuki et al., 2012; Scheen, 2014).

### Future Directions

Our current understanding is that cardiac [Na^+^]_c_ is raised in conditions of heart failure and diabetes, while SGLT2i cause the reduction of [Na^+^]_c_, through inhibition of NHE-1. Studies considering the impact of SGLT2i on NHE-1 activity, [Na^+^]_c_, [Ca^2+^]_c_, [Ca^2+^]_m_ and mitochondrial energetics in diabetic and failing cardiomyocytes are warranted. Furthermore, whether the reduction of inflammation observed with SGLT2i is a direct consequence of Na^+^ lowering is as of yet unknown. It remains uncertain whether the SGLT2 is expressed in cardiac endothelial cells and, if so, is involved in the cardiac effects of SGLT2i. Likewise, since SGLT1 expression in cardiomyocytes is increased in diabetic, heart failure and hypertrophic conditions, the effect of SGLT2i on SGLT1 under these circumstances should be investigated. The identification of SGLT2 in the endothelium with existing techniques requires accurate validation. Moreover, SMIT-1 might be a valuable target to study in the field of heart failure and SGLT2i.

## Conclusion

In conclusion, increased intracellular sodium concentration is an early hallmark and driver in the pathogenesis of heart failure and T2D. Besides other mechanisms, SGLT2i lower [Na^+^]_c_ in cardiomyocytes, activate AMPK in endothelial cells and cardiofibroblasts, and inhibit cellular glucose uptake in endothelial cells, to favorably interfere with [Ca^2+^]_c_ homeostasis, improve mitochondrial function, reduce inflammation and ROS production and restore nitric oxide formation. These effects may explain the beneficial effects of SGLT2i to prevent heart failure and other related cardiac complications in the diabetic as well as the non-diabetic heart.

## Author Contributions

LU conducted the literature review, drafted the article, provided critical revision of the article, and final approval of the version to be published. AB, CS, JF, MK, MH, RC, and NW provided critical revision of the article and final approval of the version to be published. CZ drafted the article, provided critical revision of the article, and final approval of the version to be published.

## Conflict of Interest Statement

The authors declare that the research was conducted in the absence of any commercial or financial relationships that could be construed as a potential conflict of interest.

## Abbreviations

Δ*G*_ATP_: Gibbs free energy of ATP hydrolysis
[Ca^2+^]_c_: cytoplasmatic calcium
[Na^+^]_c_: cytoplasmatic sodium
2-NBDG: 2-(N-(7-Nitrobenz-2-oxa-1,3-diazol-4-yl)Amino)-2-Deoxyglucose
AGE: advanced glycation end-product(s)
Akt: protein kinase B
AMP/ADP: adenosine monophosphate/adenosine diphosphate
AMPK: 5′ adenosine monophosphate-activated protein kinase
ASC: apoptosis-associated speck-like protein containing a caspase recruitment domain
CABG: coronary artery bypass graft
Cana: Canagliflozin
*C*_max_: maximal serum concentration
Dapa: Dapagliflozin
Drp1: dynamin-related protein
Empa: Empagliflozin
eNOS: endothelial nitric oxide synthase
ETC: electron transport chain
HAEC: human aortic endothelial cells
HbA1c: hemoglobin A1c
HFpEF: heart failure with preserved ejection fraction
HUVEC: human umbilical cord vein endothelial cells
ICAM-1: intercellular Adhesion Molecule 1
IL-1β: interleukin 1β
IL-10: interleukin 10
IL-6: interleukin 6
IR: ischemia-reperfusion
IRS-1: insulin receptor substrate 1
JNK: c-Jun N-terminal kinases
LPS: lipopolysaccharides
LTCC: L-type calcium channel
MCP-1: monocyte chemoattractant protein 1
MI: myocardial infarction
Na^+^/K^+^: ATPase sodium-potassium ATPase
NCX: Na^+^/Ca^2+^-exchanger
NFκB: nuclear factor-kappa B
NHE: sodium hydrogen exchanger
NLRP3, NACHT: LRR and PYD domains-containing protein 3
NO-cGMP-PKG: nitric oxide - cyclic guanosine monophosphate - protein kinase G
NOX2: NADPH oxidase 2
PA: palmitic acid
PCr/ATP: phosphocreatine/adenosine triphosphate
PTCA: percutaneous transluminal coronary angioplasty
ROS: reactive oxygen species
SGLT1: sodium glucose cotransporter 1
SGLT2: sodium glucose cotransporter 2
SGLT2i: sodium glucose cotransporter 2 inhibitor(s)
SMC: smooth muscle cell(s)
SMIT-1: sodium-myo-inositol co-transporter 1
SR: sarcoplasmic reticulum
STAT3: signal transducer and activator of transcription 3
STZ: streptozotocin
T2D: type 2 diabetes
TNF-α: tumor necrosis factor α
VCAM-1: vascular cell adhesion protein 1
